# Cognition in mood disorders

**DOI:** 10.1192/bjo.2020.149

**Published:** 2020-12-14

**Authors:** Katie M. Douglas, Richard J. Porter, Allan H. Young

**Affiliations:** Department of Psychological Medicine, University of Otago, New Zealand; Specialist Mental Health Services, Canterbury District Health Board, New Zealand; and Department of Psychological Medicine, University of Otago, New Zealand; Department of Psychological Medicine, Institute of Psychiatry, Psychology and Neuroscience, King’s College London, UK; and South London and Maudsley NHS Foundation Trust, Bethlem Royal Hospital, UK

**Keywords:** Bipolar affective disorders, depressive disorders, cognition, neuropsychology, mood disorders

## Abstract

Cognitive impairment plays a key role in determining the course of illness and functional outcomes in mood disorders. This article summarises and discusses important papers within this thematic series of *BJPsych Open* that contribute to a greater understanding of the complexity of ‘Cognition in Mood Disorders’.

Aspects of cognitive functioning play a vital role in determining the course of illness and functional outcome for individuals with mood disorders. Broadly speaking, cognitive functioning can be divided into emotional processing, or what has been termed ‘hot’ cognition, and non-emotional or ‘cold’ cognition. The distinction is not absolute, but in general it appears that abnormalities or biases in emotional processing may be more closely related to current mood state in mood disorders, and it is suggested that normalisation of these biases may be important for response to treatment. Cold cognition may have some relationship to mood state, particularly when this is severely perturbed, but abnormalities in these aspects of cognitive function have been found to persist into remission or euthymia. This thematic series of *BJPsych Open* addresses both aspects of cognition.

## Hot cognition

Significant evidence has accumulated regarding abnormalities in emotional processing in depression. Less clear evidence has been produced in bipolar disorder, although abnormalities in facial expression recognition and implicit emotion regulation have been more consistently found.^1^ Studies in depression have led to the cognitive neuropsychological hypothesis,^2^ which posits that negative biases in emotional processing are an important part of the pathophysiology of depression, and that successful treatments for depression reverse these, allowing more rewarding interactions and providing a positive feedback loop that continues the process of recovery from an episode of depression. According to this theory, potential treatments for depression could be screened based on whether they reverse emotional processing deficits, and if they do not, then they are unlikely to be effective antidepressants. Furthermore, the hypothesis that changes in emotional processing are a first step toward more positive social interactions and full recovery has begun to be investigated. Hobbs et al^3^ (this series) present data on the effects of citalopram in healthy volunteers in a randomised between-group study. In computerised paradigms evaluating referential affective processing, social cognition and expression of prosocial behaviour, participants who received a single dose of citalopram showed greater prosocial behaviours toward others. Exploratory analyses also suggested increased positive affective biases and increased positive recall after administration of citalopram, suggesting, as per the cognitive neuropsychological hypothesis, that there is a shift with this antidepressant toward a more positive appraisal of the environment.

Much of the research on emotional processing biases in depression and anxiety disorders has been conducted in younger people. However, with an ageing population, it is important to consider older adults, particularly given well-established data on changes in emotional processing with ageing. Gray et al^4^ (this series) examine evidence for changes in emotional processing in late-life depression and anxiety disorders. This is reviewed in light of the ‘positivity bias’ (the tendency of older adults to attend to, and remember, positive information more than negative information) in emotion processing that is observed in older adults.^5^ This might be expected to have a differential effect on emotional processing in late-life depression and anxiety compared with depression and anxiety in younger people. The review found relatively little evidence of impaired emotional processing in late-life depression, although changes in brain circuitry in this group were found to be broadly similar to those seen in younger patients. Evidence was more consistent in late-life anxiety and post-traumatic stress disorder, where there was clearer evidence of a bias toward attending to negative or threat-related stimuli, as in younger people.

## Cold cognition

Although meta-analyses have consistently suggested that impairment in cold cognition persists into euthymia,^6,7^ more recent research has focused on understanding the very significant heterogeneity in the degree of cognitive impairment seen in mood disorders.^8^ In attempting to understand this, several studies have used cluster analytic techniques to generate groups with different cognitive profiles. These have generally characterised three or four profiles, of which one has been a group of patients with relatively normal cognitive function compared with population norms or healthy controls, whereas one group has generally been characterised by global cognitive impairment. In Tsapekos et al^9^ (this series), to attempt to understand the predictors of these cognitive profiles, four separate profiles were identified in patients with bipolar disorder. Interestingly, but perhaps not surprisingly, lower cognitive reserve was associated with membership of higher impairment groups. The only other factor identified was the use of antipsychotics, which was significantly lower in the intact group.

In an attempt to understand what predicts improvement in cognitive function in the treatment of major depressive episodes, Barczyk et al^10^ (this series) examined studies that measured cognitive function at baseline and at follow-up, after treatment of an episode of major depression. Once again, age was a significant issue in the review, with studies being best understood by a split into younger and older patients. There was some evidence that increased severity of depression at baseline and increased severity of cognitive impairment at baseline both predicted a greater degree of cognitive change with treatment. These variables may be features that could be used in selecting suitable patients for future trials of, for example, cognitive remediation.

## Subjective versus objective impairment of cognitive function

It is increasingly recognised that people's subjective view of their cognitive ability may have considerable importance, even if this does not accord with results of objective cognitive testing. Research has focused on factors that make people more likely to experience subjective compared with objective impairment.^11^ For example, expectations may be important in determining subjective assessment of cognitive function. An interesting recent report in *BJPsych Open*^12^ suggested that patients who held a negative expectation of the effect that electroconvulsive therapy would have on their memory were more likely to have subjective memory worsening. Less research about subjective cognitive function has been conducted in youth depression samples, whose experience of cognitive problems associated with depression may be different if depression onset is relatively recent. In their analysis of depression symptoms over 3 months, and perceived change in subjective cognitive function, Allott et al^13^ (this series) reported that improvement or reduction in self-rated depression symptoms was highly associated with change in subjective ratings of cognitive function in their youth sample. That is, patients who reported improvement in their mood also felt their cognitive functions had strengthened. This was the case even though severity of depression in this young sample was moderate. A further interesting finding was that those with greater severity of depression at baseline were more likely to report a decline in subjective cognitive functioning over the subsequent 3 months. Much research to date has focused on objective cognitive functioning, but subjective cognitive functioning may be equally important in determining outcomes for patients.^14^ Therefore, development of interventions aimed at improving patients’ perception of cognitive functions may be helpful in enhancing recovery from mood disorders.

## Treatment of cognitive impairment

To date, there is relatively little evidence for treatments that might improve cognitive function in the group of patients for whom this is impaired long-term.^15^ However, negative trials may be explained to an extent by methodological issues. Guidelines have been written regarding appropriate methodology for cognitive enhancement trials in bipolar disorder,^16^ which have emphasised the need to screen for significant cognitive impairment and to wait until euthymia is achieved before commencing cognitive remediation strategies. Some recent studies, however, are attempting to treat cognitive impairment even when patients are still acutely depressed, with cognitive exercises hypothesised to be working by activating prefrontal brain areas.^17^ Cognitive remediation may also be useful in patients who are chronically depressed and have ongoing cognitive impairment. The field is relatively new, and it is therefore timely to produce recommendations and a review of methodology of cognitive enhancement studies in major depressive disorder. Douglas et al^18^ (this series) do this, not only examining methodologies for trials, but also attempting to give some guidance for clinicians who may wish to provide cognitive interventions for patients who are particularly impaired.

## Relationship of cognitive impairment associated with mood disorders and onset of dementia

In late-life depression and bipolar disorder, understanding cognitive function is complicated by the relationship between the mood disorders and dementia, with episodes of mood disorder being associated with risk of dementia.^19^ Barczyk et al^10^ (this series), in their review of factors that predict cognitive change, note that in some longer-term studies in late-life depression, cognitive change is predominantly negative. These studies examine what predicts a decline, finding that, for example, the number of deep white matter lesions appears to be a significant factor. A factor whose association with dementia is well established is apolipoprotein E ε4 (APOE*ε4) allele. The relationship between APOE*ε4 and depression is less clear. Burns et al^20^ (this series) showed convincing longitudinal evidence across the adult lifespan that in cognitively intact, non-depressed adults at baseline, APOE*ε4 was not associated with incident depression risk. As with in younger adult samples, further research examining cognitive trajectories in old-age depression samples would be beneficial in determining whether presence of certain factors (e.g. APOE*ε4 or deep white matter lesions) would affect cognitive remediation strategies.

## Conclusions

In summary, research examining the issue of cognitive impairment in mood disorders has revealed increasing complexity in recent years. Distinctions include between hot and cold cognition, between objective and subjective cognitive impairment, and between cognitive impairment associated with euthymia and that associated with the episodes of illness. It has become apparent that the degree of cognitive impairment is variable between individuals, with the beginnings of evidence regarding what predicts who will suffer from significant impairment. Studies have begun to examine techniques to treat cognitive impairment, but the methodological issues are complex. As patients age, this affects the profile and likely outcome of cognitive impairment associated with mood disorders. This thematic series of *BJPsych Open* touches on all these subtleties, as well as suggesting future research directions, particularly in the area of cognitive remediation.
